# Supplementary material for: In-situ incubation of a coral patch for community-scale assessment of metabolic and chemical processes on a reef slope
Source: PeerJ. 2018 Dec 3;6:e5966. doi: 10.7717/peerj.5966 (PMC6282943; doi:10.7717/peerj.5966)
Supplement: Supplemental Information 1 — ReefBudget method applied to the 4.4 m2 full planar surface of the tent. [Calcification Rate] = [Cover] * [Specific Calcification Rate] * [Rugosity] and NCCReefBudget = [Total Coral Calcification] + [Total CCA Calcification]–[Total Microbioerosion]. [file peerj-06-5966-s001.docx]

| **Species** | **Area [cm2]** | **Specific calcific. Rate** | **Cover** | **Rugosity** | **Calcification rate** |
| --- | --- | --- | --- | --- | --- |
|  | [cm2] | a) b) | c) |  | d) |
| *Meandrina meandrites* | 496 | 2.19 | 1.12 | 1.36 | 0.033 |
| *Orbicella faveolata* | 276 | 9.07 | 0.62 | 1.36 | 0.077 |
| *Siderastrea siderea* | 85 | 8.53 | 0.19 | 1.36 | 0.022 |
| *Diploria clivosa* | 756 | 4.99 | 1.71 | 1.36 | 0.116 |
| *Montastraea cavernosa* | 101 | 9.07 | 0.23 | 1.36 | 0.028 |
| *Millipora spp.* | 107 | 28.10 | 0.24 | 1.36 | 0.092 |
| *Madracis decactis* | 26 | 34.36 | 0.06 | 1.36 | 0.027 |
| **Total Coral Calcification** |  |  |  |  | **0.396** |
| **Total CCA Calcification** | 2940 | 0.18 | 17.00 | 1.36 | **0.042** |
| **Total Microbioerosion** | 9397 | 0.27 | 21.22 | 1.36 | **0.078** |
| [kg CaCO3 m^-2^ yr^-1^ planar incubation surface] |  |  |  |  | **0.360** |
| [mmol m^-2^ h^-1^ planar incubation area] |  |  |  |  | **0.411** |

| a) [kg CaCO3 m^-2^ organism yr^-1^] |
| --- |
| b) The specific calcification rates are from Perry's ReefBudget website, the 5-10 meter deep sheet; <http://geography.exeter.ac.uk/media/universityofexeter/schoolofgeography/reefbudget/documents>  /Benthic_Data_Entry_Template_5-10m_depth.xls |
| c) [% of full planar 4.4 m^2^] |
| d) [kg CaCO3 m^-2^ yr^-1^ planar incubation surface] |
