# Supplementary material for: In-situ incubation of a coral patch for community-scale assessment of metabolic and chemical processes on a reef slope
Source: PeerJ. 2018 Dec 3;6:e5966. doi: 10.7717/peerj.5966 (PMC6282943; doi:10.7717/peerj.5966)
Supplement: Supplemental Information 2 [file peerj-06-5966-s002.docx]

| Substrate classification: | Area [cm2] | % of Total | % of Hard Substrate | Source / comments |
| --- | --- | --- | --- | --- |
| Total Quadrat | 44284 |  |  | (photo survey; planar surface) |
| Total Sand | 26990 | 60.9 |  | (difference Total-Hard; planar surface) |
| Total Hard Substrate | 17294 | 39.1 |  | (photo survey; planar surface) |
| *of which...* |  |  |  |  |
| Coral | 1846 | 4.2 | 10.7 | (photo survey; planar surface) |
| Sponge | 3111 | 7 | 18 | (photo survey; planar surface) |
| CCA | 2940 | 6.6 | 17 | (photo survey; planar surface) |
| Available for bioerosion | 9397 | 21.2 | 54.3 | (difference Hard Subst. - Coral - Sponge - CCA) |
