# Supplementary material for: In-situ incubation of a coral patch for community-scale assessment of metabolic and chemical processes on a reef slope
Source: PeerJ. 2018 Dec 3;6:e5966. doi: 10.7717/peerj.5966 (PMC6282943; doi:10.7717/peerj.5966)
Supplement: Supplemental Information 3 — Rugosity. Planar areas were determined from photogrammetry, based on photographs collected after incubator removal at end of the experiment. Rugosity of 1.36 was determined in the field after removal. Rugosity of the sandy part of the patch was assumed to be approximately 1.00. Average rugosity of the patch is calculated. [file peerj-06-5966-s003.docx]

| Substrate | Planar area | Rugosity | 3D area |
| --- | --- | --- | --- |
|  | [cm2] | [cm2] | [cm2] |
| Hard | 17294 | 1.36 | 23468 |
| Sand | 26991 | 1.00 | 26991 |
| Total | 44284 | 1.14 | 50458 |
