# Supplementary material for: In-situ incubation of a coral patch for community-scale assessment of metabolic and chemical processes on a reef slope
Source: PeerJ. 2018 Dec 3;6:e5966. doi: 10.7717/peerj.5966 (PMC6282943; doi:10.7717/peerj.5966)
Supplement: Supplemental Information 4 — Rates in μmol kgSW−1 hour−1; the s.e. of the fit; and the rmses of the fits. [file peerj-06-5966-s004.docx]

| PO_4_ | 0.022±0.005 (rmse=0.009) | 0.013±0.008 (rmse=0.011) | 0.024±0.008 (rmse=0.014) | 0.045±0.014 (rmse=0.020) | 0.055±0.010 (rmse=0.020) |
| --- | --- | --- | --- | --- | --- |
| NO_2_+NO_3_ | 0.72±0.20 (rmse=0.33) | 0.82±0.31 (rmse=0.42) | 1.23±0.13 (rmse=0.22) | 1.35±0.26 (rmse=0.38) | 1.20±0.29 (rmse=0.56) |
| NH_4_ | 0.32±1.57 (rmse=2.56) | 0.38±0.70 (rmse=0.98) | 0.26±0.07 (rmse=0.11) | 0.34±0.07 (rmse=0.10) | 0.24±0.28 (rmse=0.57) |
| NO_2_ | 0.02±0.02 (rmse=0.03) | 0.02±0.02 (rmse=0.03) | 0.02±0.01 (rmse=0.01) | 0.03±0.01 (rmse=0.01) | 0.02±0.01 (rmse=0.02) |
| SIL | 0.64±0.15 (rmse=0.21) | 0.00±0.26 (rmse=0.37) | 0.28±0.15 (rmse=0.30) | -0.10±0.30 (rmse=0.46) | -0.03±0.22 (rmse=0.45) |
| DOC | 2.1±6.4 (rmse=17.8) | -1.6±6.2 (rmse=7.4) | -1.3±7.1 (rmse=13.0) | -2.8±4.1 (rmse=7.0) | -0.1±7.9 (rmse=15.4) |
| O_2_ | -13.4±NaN (rmse=3.1) | -19.9±NaN (rmse=3.3) | -20.0±NaN (rmse=2.2) | -32.0±NaN (rmse=2.7) | -25.2±NaN (rmse=1.2) |
| C_T_ | 7.4±1.1 (rmse=6.0) | 16.4±1.4 (rmse=8.3) | 19.2±1.7 (rmse=6.0) | 25.3±2.4 (rmse=3.5) | 17.3±1.8 (rmse=3.6) |
| A_T_ | 0.7±0.9 (rmse=4.3) | -3.6±1.3 (rmse=7.1) | -4.2±0.9 (rmse=5.4) | -0.6±1.2 (rmse=5.3) | 0.6±0.7 (rmse=2.4) |
| C_Tresp_ | 7.1 | 18.2 | 21.2 | 25.6 | 17.0 |
| A_Tresp_ | 0.0 | 0.0 | 0.0 | 0.0 | 0.0 |
| C_Tdiss_ | 0.3 | -1.8 | -2.1 | -0.3 | 0.3 |
| A_Tdiss_ | 0.7 | -3.6 | -4.2 | -0.6 | 0.6 |
| oxgen_const.env. | -11.3±0.2 (rmse=3.1) | -15.7±0.3 (rmse=3.7) | -17.4±0.2 (rmse=2.4) | -28.8±0.2 (rmse=2.9) | -23.9±0.1 (rmse=1.8) |
| dic_const.env. | 7.1±2.4 (rmse=6.4) | 26.8±5.4 (rmse=10.1) | 23.8±5.0 (rmse=11.4) | 30.0±2.4 (rmse=5.8) | 20.5±2.0 (rmse=5.5) |
| alk_const.env. | 3.7±1.5 (rmse=4.3) | 5.8±5.6 (rmse=12.0) | -6.4±3.3 (rmse=8.3) | 7.1±2.9 (rmse=7.5) | 2.4±1.7 (rmse=5.1) |
