# Supplementary material for: In-situ incubation of a coral patch for community-scale assessment of metabolic and chemical processes on a reef slope
Source: PeerJ. 2018 Dec 3;6:e5966. doi: 10.7717/peerj.5966 (PMC6282943; doi:10.7717/peerj.5966)
Supplement: Supplemental Information 5 — Comparison of rates of change in O2 concentrations inferred from either the full O2 concentration history (third column) or from only the first 30 minutes of each incubation (rightmost column). The latter method provides a more consistent separation between day- and night time incubations (especially when ignoring incubation 1). The daytime average O2 consumption is ∼20% below the night time rate. [file peerj-06-5966-s005.docx]

|  | time of day | full incubation | first 30 minutes |
| --- | --- | --- | --- |
| 1 | day | -11.3±0.4 | -14.2±0.2 |
| 2 | night | -15.7±0.6 | -28.4±0.5 |
| 3 | day | -17.4±0.3 | -23.1±0.5 |
| 4 | night | -28.8±0.4 | -31.5±0.6 |
| 5 | day | -23.9±0.2 | -26.2±0.4 |
