# Supplementary figures and images for: In-situ incubation of a coral patch for community-scale assessment of metabolic and chemical processes on a reef slope
Source: PeerJ. 2018 Dec 3;6:e5966. doi: 10.7717/peerj.5966 (PMC6282943; doi:10.7717/peerj.5966)

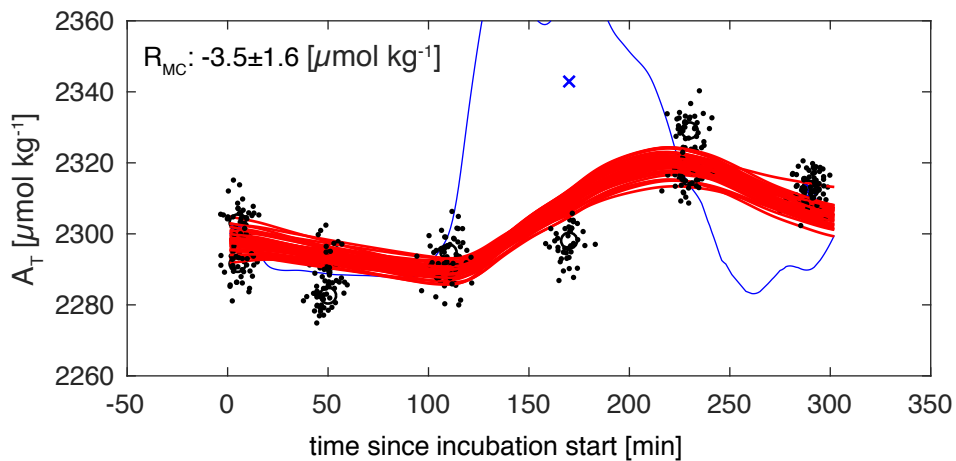

Supplement: Supplemental Information 6 — Example of results of the ‘Monte Carlo’ method used to assess robustness of inferred time rates of change of concentrations during incubation. Here, measured values of AT and the leak rate are varied slightly (±4 μmol kg−1 for AT, ±0.1 % min−1 for leak rate), and curves are repeatedly fit. The average and standard deviation of one thousand such fits are presented. In this example, the rate average is slightly larger than the associated uncertainty, and the rate is thus assumed to be significantly different from zero. [file peerj-06-5966-s006.pdf]

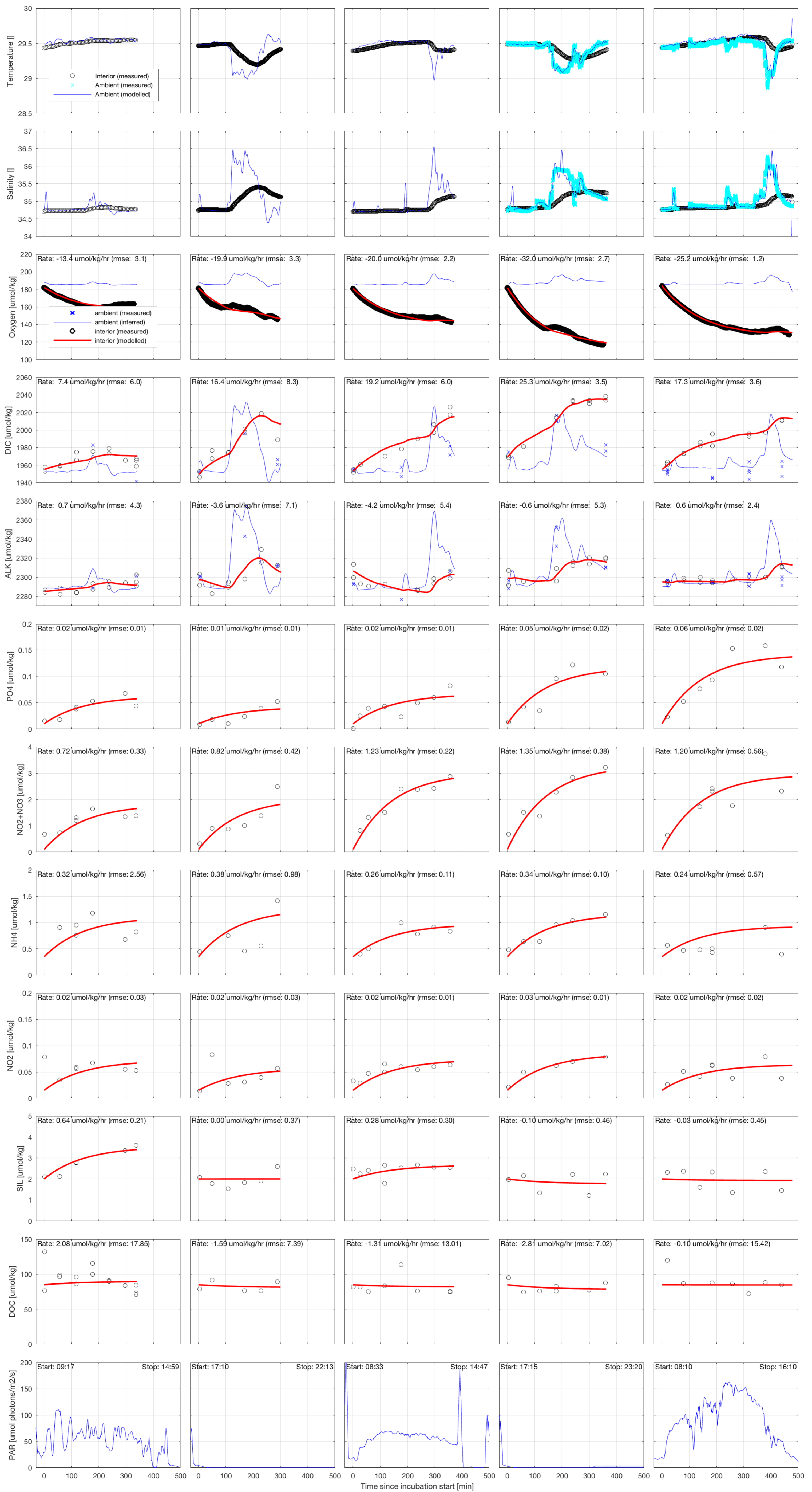

Supplement: Supplemental Information 8 — In-tent measurements and model fits of all five incubation periods. Second and fourth incubations were during nighttime. The legend presented in the third row of panels applies to all subsequent rows (except PAR, which was measured). [file peerj-06-5966-s008.pdf]

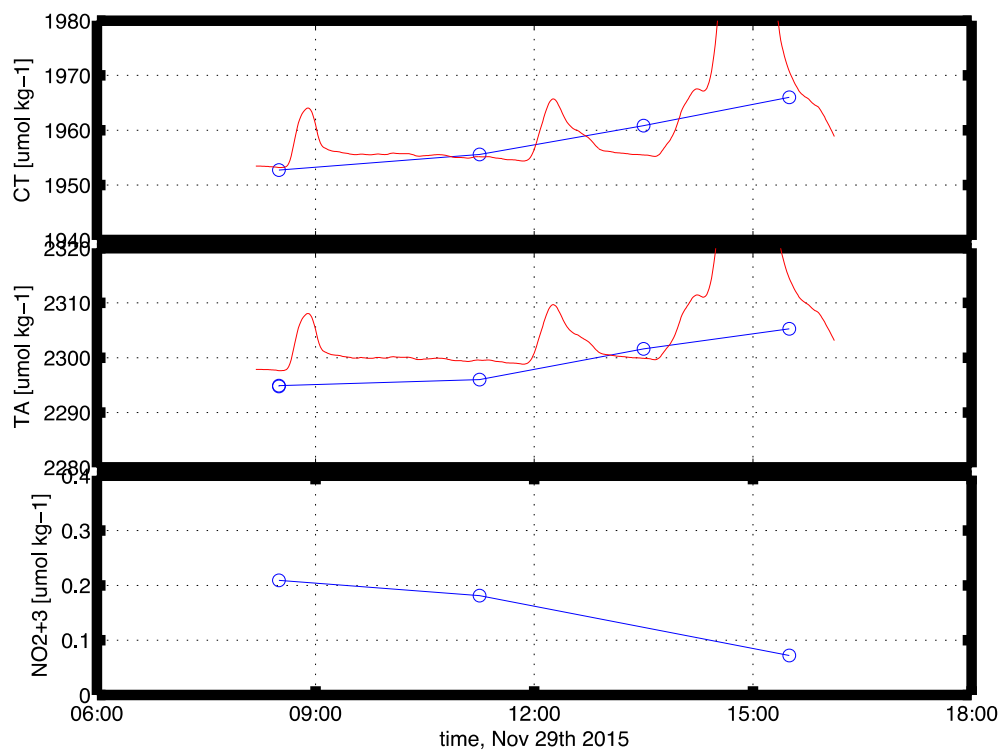

Supplement: Supplemental Information 9 — Data from secondary ‘pyramid’ incubator, placed on bare sediment. The observed trends are the result of the (unknown) balance between (i) exchange with the environment and (ii) sedimentary processes. Irrespective of dominant process, sedimentary fluxes are inferred to be negligibly low compared to those observed in the primary incubator (see main text). [file peerj-06-5966-s009.pdf]
