# Supplementary material for: In-situ incubation of a coral patch for community-scale assessment of metabolic and chemical processes on a reef slope
Source: PeerJ. 2018 Dec 3;6:e5966. doi: 10.7717/peerj.5966 (PMC6282943; doi:10.7717/peerj.5966)
Supplement: Supplemental Information 7 — Interior and ambient nutrient concentrations. [file peerj-06-5966-s007.pdf]

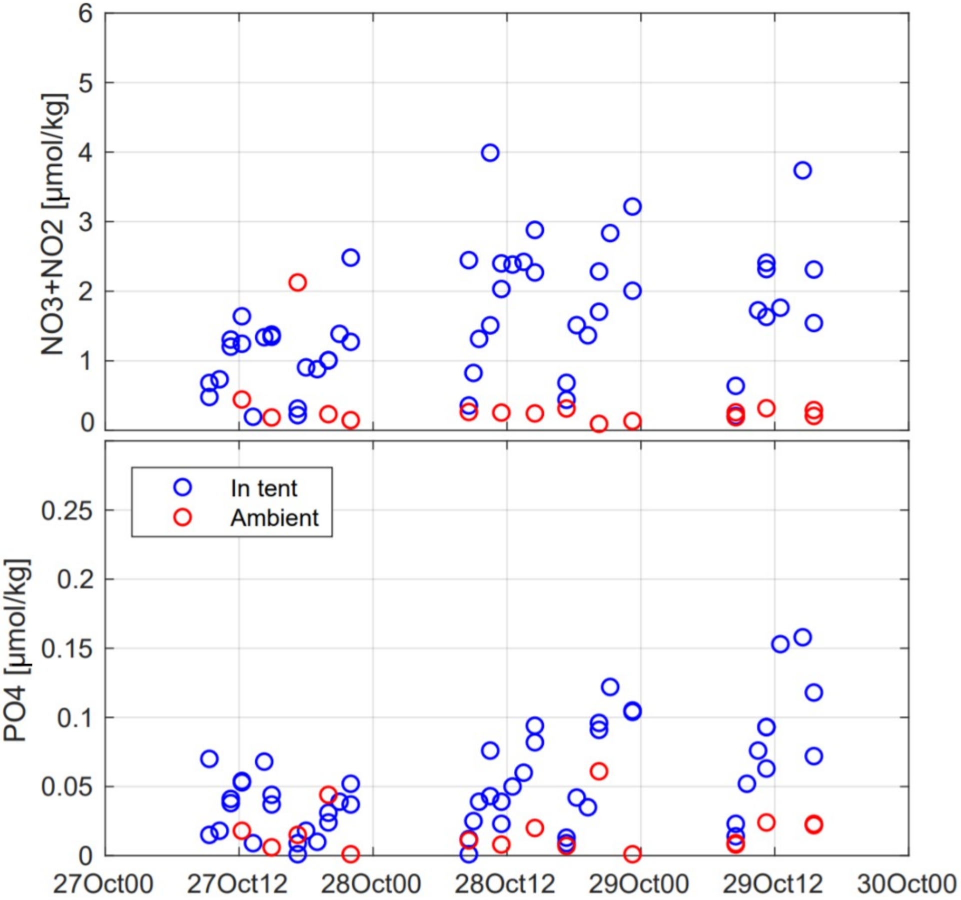

NO3+NO2 [μmol/kg]
6
5
4
3
2
1
0
PO4 [μmol/kg]
0.25
0.2
0.15
0.1
0.05
0
In tent
Ambient
27Oct00
27Oct12
28Oct00
28Oct12
29Oct00
29Oct12
30Oct00
